# Generating induced pluripotent stem cells from common marmoset (*Callithrix jacchus*) fetal liver cells using defined factors, including Lin28

**DOI:** 10.1111/j.1365-2443.2010.01437.x

**Published:** 2010-09

**Authors:** Ikuo Tomioka, Takuji Maeda, Hiroko Shimada, Kenji Kawai, Yohei Okada, Hiroshi Igarashi, Ryo Oiwa, Tsuyoshi Iwasaki, Mikio Aoki, Toru Kimura, Seiji Shiozawa, Haruka Shinohara, Hiroshi Suemizu, Erika Sasaki, Hideyuki Okano

**Affiliations:** 1 Central Institute for Experimental AnimalsKanagawa, Japan; 2 School of Medicine, Keio UniversityTokyo, Japan; 3 JAC Inc.Tokyo, Japan; 4 Genomic Science Laboratories, Dainippon Sumitomo Pharma Co. LtdOsaka, Japan; 5 PRESTO Japan Science and Technology AgencyTokyo, Japan

## Abstract

Although embryonic stem (ES) cell–like induced pluripotent stem (iPS) cells have potential therapeutic applications in humans, they are also useful for creating genetically modified human disease models in nonhuman primates. In this study, we generated common marmoset iPS cells from fetal liver cells via the retrovirus-mediated introduction of six human transcription factors: Oct-3/4, Sox2, Klf4, c-Myc, Nanog, and Lin28. Four to five weeks after introduction, several colonies resembling marmoset ES cells were observed and picked for further expansion in ES cell medium. Eight cell lines were established, and validation analyses of the marmoset iPS cells followed. We detected the expression of ES cell–specific surface markers. Reverse transcription-PCR showed that these iPS cells expressed endogenous *Oct-3/4*, *Sox2*, *Klf4*, *c-Myc*, *Nanog* and *Lin28* genes, whereas all of the transgenes were silenced. Karyotype analysis showed that two of three iPS cell lines retained a normal karyotype after a 2-month culture. Both embryoid body and teratoma formation showed that marmoset iPS cells had the developmental potential to give rise to differentiated derivatives of all three primary germ layers. In summary, we generated marmoset iPS cells via the transduction of six transcription factors; this provides a powerful preclinical model for studies in regenerative medicine.

## Introduction

A report on induced pluripotent stem (iPS) cells in 2006 generated wide interest in cell reprogramming using defined factors (Takahashi & Yamanaka 2006). Via the retroviral introduction of four transcription factors, Oct-3/4, Sox2, Klf4, and c-Myc, adult mouse fibroblasts were reprogrammed to an undifferentiated state similar to that of embryonic stem (ES) cells. This was followed by a report that the new iPS cells are able to generate chimeric mice with high efficiency, to contribute to germ cell development, and to produce iPS progeny mice through germline transmission using the Nanog reporter (Okita *et al.* 2007). Five independent groups established human iPS cells (Masaki *et al.* 2007; Takahashi *et al.* 2007; Yu *et al.* 2007; Lowry *et al.* 2008; Park *et al.* 2008), and this was followed by the establishment of rhesus monkey iPS cells (Liu *et al.* 2008). Four of these groups used Oct-3/4, Sox2, Klf4, and c-Myc to reprogram somatic cells, whereas Thomson’s group (Yu *et al.* 2007) used Oct-3/4, Sox2, Nanog, and Lin28. Nonhuman primate iPS cells have great potential for use in research on human diseases through models of human diseases. iPS cell technology has progressed dramatically in recent years and now promises not only the development of regenerative medicine for humans but also significant improvements in the generation of genetically modified animals for biomedical purposes. Yet to realize the potential of regenerative medicine using iPS cells, several problems must be resolved. One of the most critical is evaluating the safety of using iPS cells in human patients. Nothing is known of the tumorigenesis of transplanted iPS cell-derived cells via autografts or allografts in humans because human iPS cell-derived cells have only been transferred into immune-deficient mice, which have high oncogenicity. Furthermore, the life span of mice is too short to assess the long-term side effects of regenerative medicine using human iPS cells. Thus, ideally, the therapeutic efficacy of the transplantation of differentiated human iPS cells must be elucidated using experimental animals more closely related to humans through autogenic or allogenic transplantation.

The common marmoset (*Callithrix jacchus*), a nonendangered New World primate that is native to Brazil, offers many advantages over other laboratory primates for use in medical studies, including studies of reproductive biology. It is an excellent model of human disease because its neurophysiological functions, metabolic pathways, and drug sensitivities are similar to those of humans. Moreover, recently, we reported the creation of the first transgenic marmosets with germline transmission through the lentiviral vector–mediated gene transfer (Sasaki *et al.* 2009), after the establishment of common marmoset ES cells in 2005 (Sasaki *et al.* 2005). Hence, the establishment of iPS cells in this species will dramatically accelerate the development of preclinical studies of regenerative medicine. If marmoset iPS cells are produced, a very precise system for assessing the safety and efficacy of regenerative medicine could be established. Using this system, both human and marmoset iPS cell-derived cells could be transplanted into marmosets of human disease model such as spinal cord injury (Iwanami *et al.* 2005), and this system could be used to evaluate the effects on animals of both major histocompatibility complex–matching allogenic transplantation (marmoset iPS cell-derived cells) and xenogenic transplantation (human iPS cell-derived cells). In this study, we established common marmoset iPS cells and characterized their differentiation capacity.

## Results

### Generation of marmoset iPS cells from fetal liver cells

**Figure 1:**
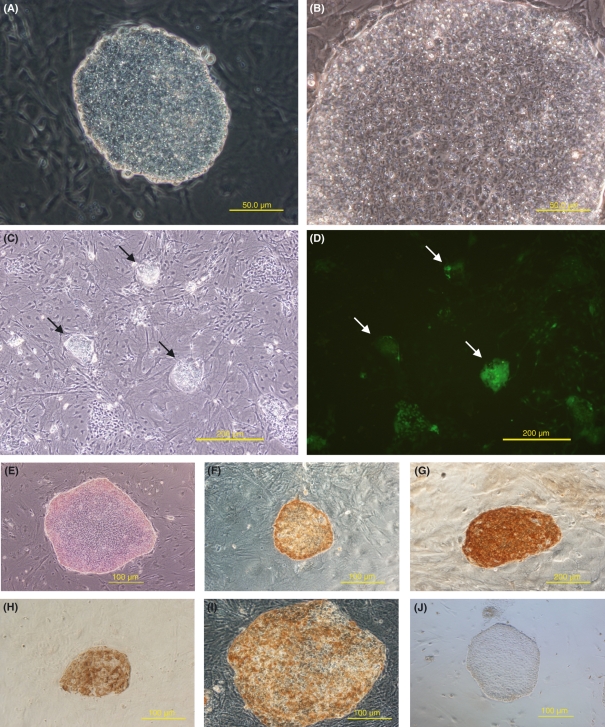
Four to five weeks after we introduced six transcription factors, induced pluripotent stem (iPS) colonies resembling embryonic stem cells in morphologically were observed (A and B). All iPS cells had a flat, packed, tight colony morphology and a high nucleus-to-cytoplasm ratio (B). Most of the other various types of iPS cell colonies (C) expressed green-fluorescent protein fluorescence (D). The iPS cells (line A) showed alkaline phosphatase activity (E) and expressed SSEA-3 (F), SSEA-4 (G), TRA-1-60 (H), and TRA-1-81 (I) but not SSEA-1 (J).

In our preliminary studies, we did not produce cells that met the criteria of iPS cells using Yamanaka’s four transcription factors (Oct4, Sox2, Klf4, and c-Myc). Therefore, we used six transcription factors to generate marmoset iPS cells: Yamanaka’s four factors plus Nanog and Lin28. Four to five weeks after we introduced the six transcription factors into marmoset fetal liver cells, we observed colonies resembling ES cells morphologically (Fig. 1A,B), consistent with the report on rhesus monkey iPS cells (Liu *et al.* 2008). All of the observed colonies that resembled marmoset ES cells were green-fluorescent protein (GFP)-negative, whereas most of the other colonies with different morphologies were GFP-positive (Fig. 1C,D). Picked-up colonies were plated on an irradiated mouse embryonic fibroblasts (MEFs) feeder layer, and 8 of 25 cell lines were cultured for more than 10 passages (18.3% derivation rate). Microsatellite analysis showed a complete genotypic match between the original liver cells and generated iPS-like cells (Fig. S1 in Supporting Information), showing that these iPS-like cells were certainly generated from liver cells. All of the iPS-like cells had a flat, packed, and tight colony morphology and a high nucleus-to-cytoplasm ratio (Fig. 1B), and continuous cultures of iPS-like cells have been sustained for more than 9 months.

### Characterization of marmoset iPS cells

**Figure 2:**
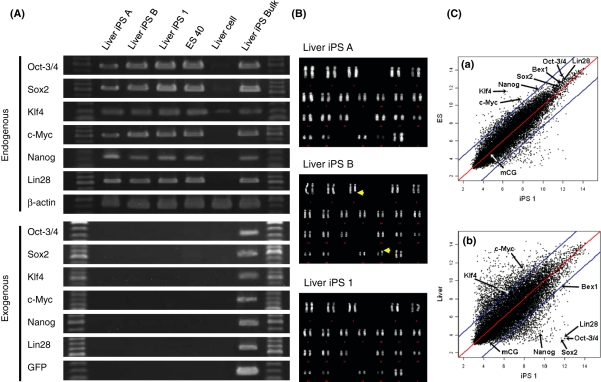
(A) RT-PCR showed that all of the induced pluripotent stem (iPS) cell lines also expressed endogenous *Oct-3/4*, *Sox2*, *Klf4*, *c-Myc*, *Nanog* and *Lin28* genes, whereas all of the transgenes were silenced. Bulk liver iPS cells referred to all of the cells generated from the cells into which transgenes were introduced. Therefore, bulk liver iPS cells contained both cells expressing transgenes and cells with silenced transgenes. (B) Two iPS cell lines (Liver iPS A and 1) retained the normal 46, XX karyotype after a 2-month culture, whereas one line (Liver iPS B) showed translocation between chromosomes 3 and 22 (arrow head). (C) (a) Custom DNA microarray Marmo2 was used to compare global expression patterns between marmoset iPS cells (Liver iPS 1) and marmoset embryonic stem cells. (b) Global expression patterns were compared between marmoset iPS cells (Liver iPS 1) and the original fetal liver cells. The red line indicates the diagonal, and the blue lines indicate fivefold changes between the two samples.

To confirm the undifferentiated status of the iPS-like cells, we examined the expression of ES cell–specific cell surface markers on three lines (iPS A, B, 1) in which six transcription factors had been introduced. As shown in Fig. 1 and Fig. S2 in Supporting Information, the iPS-like cell lines showed alkaline phosphatase activity (Fig. 1E) and expressed SSEA-3 (Fig. 1F), SSEA-4 (Fig. 1G), TRA-1-60 (Fig. 1H), and TRA-1-81 (Fig. 1I) but not SSEA-1 (Fig. 1J). Reverse transcription (RT)-PCR showed that these three iPS-like cell lines also expressed endogenous Oct4, Sox2, Klf4, c-Myc, Nanog, and Lin28 genes, whereas all of the transgenes were silenced (Fig. 2A). Two iPS-like cell lines (iPS A and 1) retained the normal 46, XX karyotype (Fig. 2B) after a 2-month culture, whereas one line (iPS B) showed translocation between chromosomes 3 and 22. Microarray analyses showed that the global gene expression patterns were similar but not identical between marmoset iPS cells and ES cells (Fig. 2C). In contrast, the patterns showed substantial differences between marmoset iPS cells and the original fetal liver cells (Fig. 2C). There were strong similarities between the iPS cells and ES cells in the markers of undifferentiated states, such as Oct-3/4, Sox2, Nanog, and Lin28 (Fig. S3 in Supporting Information). From these results, we concluded that these cell lines were marmoset iPS cells.

### *In vitro* differentiation of marmoset iPS cells

**Figure 3:**
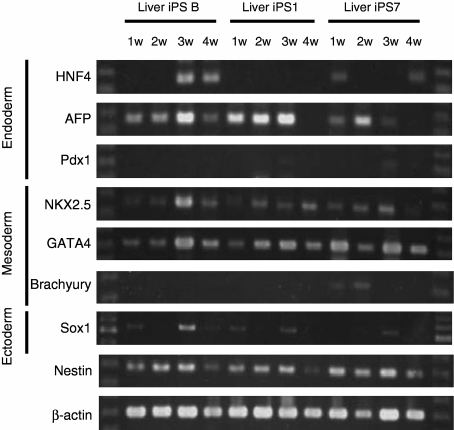
In all induced pluripotent stem (iPS) cell lines, the *Nkx2.5*, *GATA4*, and n*estin* genes were expressed continually throughout the 4-week culture, and the *brachyury*, *HNF4*, *AFP*, and *Sox1* genes were partially expressed in several iPS cell lines, showing that all iPS cells had the developmental potential to give rise to differentiated derivatives of all three primary germ layers.

**Figure 4:**
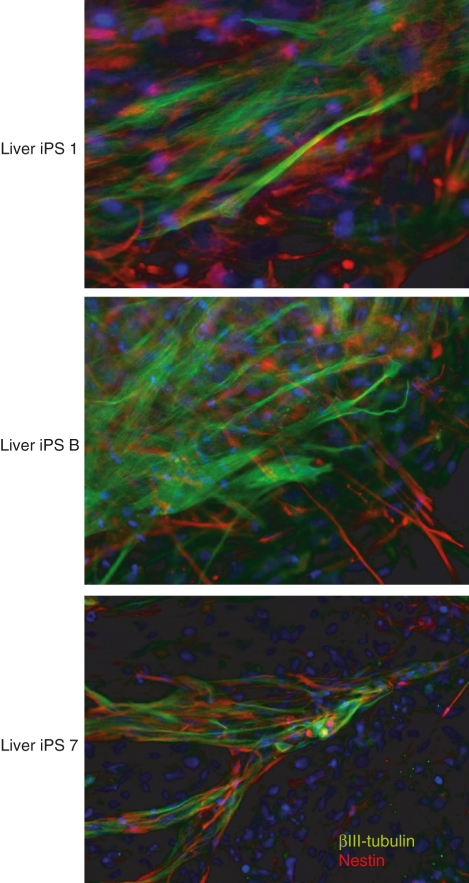
After a 24-day culture on PA6 cells, cells positive for the neural progenitor marker Nestin (red) and neuronal marker βIII-tubulin (green) were observed.

To assess the spontaneous differentiation potency of the established iPS cells *in vitro*, we examined the formation of embryoid bodies (EBs) and the expression of several genes. Suspension cultures of all three iPS cell lines formed EBs. Simple EBs formed several days after the start of the suspension cultures, and cystic EBs formed within 2 weeks. To examine the gene expression of these EBs, we used primers for the *Nkx2.5*, *GATA4*, *brachyury*, *HNF4*, *AFP*, *Pdx1*, *nestin*, and *Sox1* genes, which are marker genes for the three germ layers. The *Nkx2.5*, *GATA4*, and *nestin* genes were expressed continually throughout the 4-week culture, and the *brachyury*, *HNF4*, *AFP*, and *Sox1* genes were partially expressed (Fig. 3), showing that all of the iPS cells had the developmental potential to give rise to differentiated derivatives of all three primary germ layers. Moreover, spontaneous beating cells were found on *in vitro* culture, showing the capacity for differentiation into cardiomyocytes. We used the modified stromal cell-derived inducing activity (SDIA) method (Kawasaki *et al.* 2000) to further investigate the *in vitro* differentiation potency of marmoset iPS cells into neural cells. After a 24-day culture on PA6 cells, cells positive for the neural progenitor marker Nestin and neuronal marker βIII-tubulin were observed in all of the iPS cell lines tested (Fig. 4).

### Teratoma formation of marmoset iPS cells

**Figure 5:**
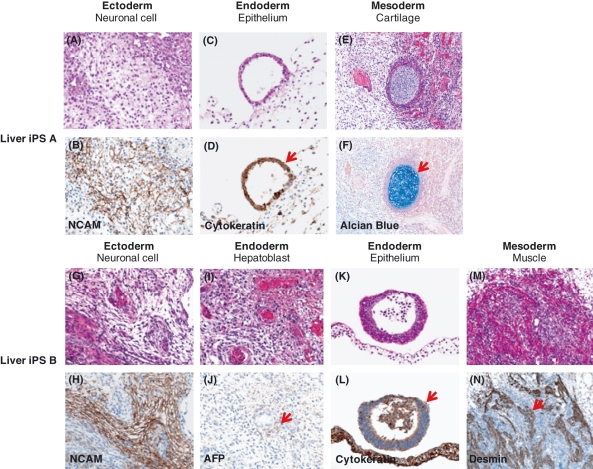
As evidence of induced pluripotent stem cell differentiation into ectodermal cells, the NCAM-positive cells were observed as neuronal cells (A, B, G, and H). The presence of the columnar epithelium (C, D, K, and L) consisting of cytokeratin-positive cells and hepatoblasts (I and J) that were AFP-positive cells suggests endodermal differentiation. The muscle-like structure expressed desmin (M and N). Alcian Blue staining showed cartilage (E and F).

To examine the differentiation potency in more detail, we injected cells of three iPS cell lines into capsules of the kidneys of NOD/shi-scid and IL-2Rγnull (NOG) mice (Ito *et al.* 2002). Eight weeks after injection, tumors under the capsules were removed from these mice and subjected to histologic analysis. The tumors were found to be teratomas that consisted of embryonic germ layers of ectodermal, mesodermal, and endodermal tissues (Fig. 5). Differentiation was confirmed by immunohistochemical analysis with several tissue-specific antibodies. As evidence of the differentiation of iPS cells into ectodermal cells, the neural cell adhesion molecule (NCAM)-positive cells were observed to be neuronal cells (Fig. 5A,B,G,H). The presence of the columnar epithelium (Fig. 5C,D,K,L), which consisted of cytokeratin-positive cells, and hepatoblasts (Fig. 5I,J), which consisted of AFP-positive cells, suggested endodermal differentiation. The teratomas differentiated frequently into mesodermal tissues, such as muscle, blood vessels, and cartilage. The muscle-like structure expressed desmin (Fig. 5M,N), and Alcian Blue staining showed cartilage (Fig. 5E,F).

### Optimization of the indispensable genes for generating marmoset iPS cells

Because no iPS cell line that met the criteria of iPS cells was established after we had introduced Yamanaka’s four transcription factors, we tried to generate iPS cells using Nanog plus those four factors (Nanog 5-factors) or Lin28 plus those four factors (Lin28 5-factors) to determine whether Nanog or Lin28 was a critical factor. Three iPS cell lines of 11 colonies resembling ES cells morphologically were cultured for more than 10 passages when we used Lin28 5-factors, whereas no iPS cell line was established when we used Nanog 5-factors.

## Discussion

In this study, we established common marmoset iPS cells from fetal liver cells via the retrovirus-mediated introduction of six human transcription factors: Oct-3/4, Sox2, Klf4, c-Myc, Nanog, and Lin28. We first attempted to generate marmoset iPS cells with Yamanaka’s four factors using adult or newborn skin fibroblasts and bone marrow cells. As a result, we obtained several marmoset iPS cell lines that resembled mouse-type ES cells morphologically (i.e., small, round and tightly aggregated) and that expressed all of the undifferentiated markers. However, they were ‘incomplete’ iPS cells because transgene silencing was not observed (data not shown). Moreover, we could not obtain ‘complete’ iPS cells that show the transgene silencing and primate ES cell–like morphology from those cellular sources even when we used the six transcription factors. In contrast, when we introduced the six transcription factors into fetal liver cells, colonies resembling marmoset ES cells emerged 4–5 weeks after introduction and their GFP fluorescence was negative in all colonies, indicating the silencing of exogenously introduced transgenes. Because all transgenes were silenced in iPS cell colonies resembling marmoset ES cells from the results of RT-PCR and most of the other ‘incomplete’ colonies were GFP-positive, transgene silencing seems to be an essential requirement for the generation of ‘complete’ iPS cells. The GFP fluorescence of the liver cells that were infected with the transgenes disappeared approximately 2–4 weeks after transgene infection. Because liver cells have a greater tendency to silence the transgenes than do bone marrow cells, we successfully produced iPS cells in this study.

We also attempted to generate marmoset iPS cells from fetal liver cells, but use of Yamanaka’s four transcription factors did not produce ‘complete’ iPS cells. Hence, we tried to generate iPS cells using Nanog 5-factors or Lin28 5-factors to determine whether Nanog or Lin28 is the critical factor. The fact that iPS cells were generated from the cells into which Lin28 5-factors were introduced indicates that Lin28 in addition to those four factors play an important role in generating iPS cells in the common marmoset. Notably, only one iPS cell colony resembling marmoset ES cells was observed after we introduced Nanog 5-factors, although we could not maintain that colony. It is clear that Lin 28 in addition to Yamanaka’s four transcription factors increases the probability of iPS cell generation in the common marmoset. In fact, Lin28 is shown to play crucial roles as a reprogramming factor for generating human iPS cells by Thomson’s group (Yu *et al.* 2007), because they discovered that primates ES cells strongly expressed Lin28. Among experimental animals, primates have a great deal of potential as nonhuman models of human disease, and the same holds for primates iPS cells. Hence, there is a need to develop a methodology for generating primates iPS cells without viral integration to avoid the risk of tumorigenicity. Several improved methods for generating iPS cells without the integration of transgenes have been reported using nonviral plasmid vectors (Okita *et al.* 2008), adenovirus vectors (Stadtfeld *et al.* 2008), the cre-loxP system (Kaji *et al.* 2009), the piggyBac system (Woltjen *et al.* 2009), and Sendai virus vectors (Fusaki *et al.* 2009). Although the use of several small molecules and chemicals in combination with several transgenes improves the efficiency of iPS cell generation, the efficiency of iPS cell derivation is markedly lower than when viral integration is used. Consequently, use of the Lin28 transgene in addition to other factors or methods might be an efficient method for generating primate iPS cells.

Wu *et al.* (2010) recently reported the generation of marmoset iPS cells, although the *in vitro* differentiation abilities of these cells have not been characterized. In our study, the *in vitro* indirect or direct differentiation abilities of marmoset iPS cells were shown by EB formation or modified SDIA methods. These results indicated that marmoset iPS cells can be used in preclinical studies of regenerative medicine, especially in models of major histocompatibility complex–matching allogenic transplantation. There are methodological differences between a recent report by Wu *et al.* (2010) and this study. They infected retroviruses carrying four transcription factors (Oct3/4, Klf4, Sox2 and cMyc) for three times and used a HDAC inhibitor (valproic acid) during reprogramming processes. Large number of retrovirus infections might cause strong epigenetic modifications by the chromosomal integration of retroviruses, and the use of HDAC inhibitor might generate chromosomal instability (Sakurada 2010). To apply the iPS cells to medical use, we must avoid the methodologies that have risks of tumorigenesis as much as possible and preclinical studies using nonhuman primate should be carried out sufficiently through allogenic transplantation. Based on these grounds, establishment of marmoset iPS cells with six transcription factors in this study would be meaningful and valuable.

Same as in a previous report on common marmoset ES cells (Sasaki *et al.* 2005), iPS cell colonies maintained a tight and packed morphology and had many similarities to human and other nonhuman primate ES cells in terms of morphology, surface antigens, and cellular characteristics (Thomson *et al.* 1995, 1998; Reubinoff *et al.* 2000; Suemori *et al.* 2001). They maintained an undifferentiated state expressing endogenous Oct-3/4, Sox2, Klf4, c-Myc, Nanog, and Lin28, and showed a normal ability to differentiate into three germ cell layers both *in vitro* and *in vivo* that was equivalent to that of marmoset ES cells (Sasaki *et al.* 2005). Microarray analyses showed that the global gene expression patterns were similar between marmoset iPS cells and ES cells. Specifically, the expression level of mCG and Bex1, which reflect the ability of primate ES cells to differentiate into trophectodermal cells, were almost the same in marmoset iPS and ES cells (Fig. 2C), showing that marmoset iPS cells are incapable of chimeric formation just like other primates ES cells or mouse epiblast stem cells.

In contrast, as reported for human ES and iPS cells (Chin *et al.* 2009), slight differences of gene expression patterns were observed between iPS cells and ES cells (Fig. 2 and Fig. S3 in Supporting Information). These differentially expressed genes between iPS and ES cells are referred as ‘reprogramming-recalcitrant’ genes (Sakurada 2010), as they resist the induction of transcriptional state identical to that seen in ES cells mainly because of (i) insufficient induction of ES cell–specific genes; (ii) insufficient suppression of somatic cell–specific genes; and (iii) induction of iPS cell–specific genes. At least the first mechanism could be relevant to the present observation, as the expression level of Nanog was somewhat higher in marmoset ES cells than in marmoset iPS cells, although Nanog expression got higher in iPS cells compared to original fetal liver cells (Fig. 2C). In the case of mouse cells, we found that mouse iPS-derived neural stem/progenitor cells showed substantially varied teratoma-forming propensities depending the iPS cells’ tissue of origin, whereas the teratoma-forming propensity of mouse ES-derived neural stem/progenitor cells was very low in the same condition (Miura *et al.* 2009). Thus, identification of ‘reprogramming-recalcitrant’ genes is crucial in the evaluation of the safety issues. Based on these findings, the identifications of functional difference caused by the different gene expression profile of marmoset iPS cells and ES cells as well as differences in reprogramming recalcitrant genes between human iPS cells and marmoset iPS cells would be important for considering their preclinical applications. These issues will be addressed in our future investigations.

Over the past few decades, numerous studies of reproductive biology have involved marmosets. The availability of marmosets and their ease of breeding suggest that these primates represent a promising alternative to more traditional old-world nonhuman primates. In the future, common marmosets and their iPS cells will provide a powerful preclinical model for studies in the field of regenerative medicine, and this should lead to a surge of interest among biologic researchers.

## Experimental procedures

### Animals

Common marmosets have been maintained in cages measuring 39 × 60 × 70 cm in our laboratory at the Central Institute for Experimental Animals (CIEA) since 1975. This study was approved by the animal ethics committees and gene recombination experiment safety management committees of CIEA and was performed in accordance with CIEA guidelines.

### Cell culture

Fetal liver cells were isolated from a miscarried female fetus. The liver was removed using iris scissors and washed twice. After mincing with iris scissors on a 60-mm culture dish, the minced tissues were dissociated in 0.25% trypsin-EDTA solution (Invitrogen, Carlsbad, CA, USA) at 37 °C for 15 min. The trypsinized cells were washed once by centrifugation at 190 ***g*** for 5 min and subsequently seeded into 100-mm plastic culture dishes. The seeded cells were cultured for 6–8 days in Dulbecco’s Modified Eagle Medium (DMEM; Invitrogen) supplemented with 10% fetal bovine serum (FBS; JRH, Tokyo, Japan) and 1% Antibiotic-Antimycotic (Invitrogen) at 37 °C in a humidified atmosphere of 5% CO_2_ and 95% air. After we removed unattached clumps of cells or explants, the attached cells were further cultured until confluent, subcultured at intervals of 5- to 7-day intervals by trypsinization for 5 min using trypsin-EDTA solution, and stored after two passages in freezing medium (Cell Banker; Mitsubishi Chemical Medience Corp., Tokyo, Japan) at −80 °C.

### Retroviral production of human six factors

Retroviral pMX vectors for human Oct-3/4, Sox2, Klf4, c-Myc, Nanog, and Lin28 were kindly provided by Dr Yamanaka (Takahashi *et al.* 2007). Retroviruses of these transcription factors were produced using the ‘Retroviral Gene Transfer and Expression System’ (Takara Bio Inc., Shiga, Japan) according to the manufacturer’s instructions. Briefly, GP-2 cells were plated at 3 × 10^6^ cells per 100-mm dish and incubated overnight. The cells were transfected with 6 μg of pMX vectors with 6 μg of pVSV-G vectors by FuGENE 6 transfection reagent (Roche, Basel, Switzerland), followed by replacement with a new medium the next day. The medium was collected 48 and 72 h after transfection as a virus-containing supernatant and filtered through a 0.45-μm-pore cellulose acetate filter (Sartorius, Göttingen, Germany). Virus stocks were stored at −80 °C until use.

### Retroviral infection and iPS cell generation

Common marmoset fetal liver cells were seeded at 1 × 10^6^ cells per 10-cm dish 1 day before transduction. The medium was replaced with virus-containing supernatant supplemented with 4 μg/mL Polybrene (Nacalai Tesque, Kyoto, Japan), and incubated for 12 h. Seven days after introduction, the cells were harvested by trypsinization and plated onto MEFs at 1 × 10^5^ cells per 10-cm dish. At the same time, the DMEM containing 10% FBS was replaced with medium for ES cell culture that consisted of Knockout DMEM supplemented with 10% Knockout Serum Replacement (KSR; Invitrogen), 1 mm l-glutamine, 0.1 mm MEM nonessential amino acids, 0.1 mmβ-mercaptoethanol (2-ME; Sigma-Aldrich Japan, Tokyo, Japan), 1% Antibiotic-Antimycotic, and 10 ng/mL leukemia inhibitory factor (LIF; Millipore, Billerica, MA, USA). The medium was changed every other day. Three to five weeks after we introduced the transgenes, colonies were picked up and dissociated mechanically into small clamps by pipetting up and down. The cell suspension was transferred onto MEFs in 12-well plates and cultured in 0.2 mL medium for ES cell culture. For cell splitting, undifferentiated iPS cell colonies were detached from the feeder cells using 0.25% trypsin supplemented with 1 mm CaCl_2_ and 20% KSR. The removed colonies were dissociated mechanically into 10–50 cells and replated on a new irradiated MEF feeder layer.

### Immunohistochemical staining

To examine the expression of cell-surface markers on cultured marmoset iPS cells, we detected alkaline phosphatase using the Alkaline Phosphatase Detection kit (Millipore) according to the manufacturer’s instructions. Immunostaining followed a previous report (Sasaki *et al.* 2005). Briefly, ES cells were fixed with 4% paraformaldehyde in phosphate-buffered saline (PBS) for 10 min at room temperature and then incubated with 0.3% H_2_O_2_ for 10 min at room temperature. The primary antibodies against stage-specific embryonic antigen (SSEA)-1, SSEA-3, SSEA-4 (Developmental Studies Hybridoma Bank, Iowa City, IA, USA), TRA-1-60, and TRA-1-81 (Millipore) were diluted with Antibody Diluent (DAKO ChemMate; DakoCytomation, Glostrup, Denmark) and incubated for 1 h at room temperature. The following primary antibodies (dilutions) were used: anti-SSEA-1 (1 : 100), anti-SSEA-3 (1 : 100), anti-SSEA-4 (1 : 200), anti-TRA-1-60 (10 μg/mL), and anti-TRA-1-81 (10 μg/mL). After three washes with PBS, the biotinylated secondary antibody Simple Stain MAX-PO Multi system (Nichirei Corporation, Tokyo, Japan) was incubated with the cells for 30 min at room temperature. The samples were washed three times with PBS, and the bound monoclonal antibodies were located using the DAB (3,3′-diaminobenzidine tetrahydrochloride) horseradish peroxidase complex.

For immunohistochemical analysis of tumors that formed after transplantation into immunodeficient mice, the collected tumors were fixed in neutral-buffered formalin and embedded in paraffin. The paraffin blocks were sectioned and subjected to immunohistochemical staining. Primary antibodies against cytokeratin, desmin, NCAM, and AFP (all purchased from DakoCytomation) were incubated with the paraffin sections. The localization of the bound monoclonal antibodies was detected using the Envision System (DakoCytomation). Several sections were stained with Alcian Blue.

### Karyotypic analysis

All iPS cell lines were subjected to karyotype analysis using a modification of the Q-banding technique (Sugawara *et al.* 2006). Briefly, iPS cells were incubated with medium containing 100 ng/mL colcemid (Invitrogen) for 90 min. Then they were then washed in PBS, dissociated using trypsin-EDTA solution, and spun down. The chromosome slides were stained with 0.01 μg/mL Hoechst 33258 for 5 min and then with 5.0 g/mL quinacrine mustard for 20 min. The stained slides were mounted in a mount solution for observation through a Leica DMRXA2 fluorescent microscope and analyzed using Leica CW4000.

### RT–PCR

RNA was isolated using the RNeasy mini kit (QIAGEN, Valencia, CA, USA) according to the manufacturer’s instructions. First-strand cDNA was synthesized from RNA from undifferentiated ES cells or EBs using the QuantiTect Reverse Transcription kit (QIAGEN). As a negative control, RNA was allowed to react with the cDNA synthesis reaction mixture in the absence of reverse transcriptase. After cDNA synthesis, the cDNA synthesis reaction mixture was used as the template for the PCR. The primers used are shown in Fig. S4 in Supporting Information. The PCR reaction mixture (20 μL) contained ×1 PCR buffer [10 mm Tris–HCl (pH 9.0), 1.5 mm MgCl_2_, 50 mm KCl], 0.2 mm dNTP, 0.5 μm of each primer, and 2.5 U *Taq* polymerase. The amplification was performed for 35 cycles of 98 °C for 20 s, 60 °C for 25 s, and 72 °C for 25 s.

### EB formation

To study EB formation, we removed undifferentiated iPS cells from the MEF feeder layer, dissociated them using 0.25% trypsin in PBS with 20% KSR and 1 mm CaCl_2_, and cultured them in bacterial Petri dishes for 7–28 days using DMEM supplemented with 10% FBS. The medium was changed every 2 days.

### *In vivo* differentiation: teratoma formation

To examine teratoma formation in mice, we injected 1–5 × 10^6^ iPS cells into the capsules of the kidneys of 5-week-old immunodeficient NOG mice (Ito *et al.* 2002). Four to eight weeks after the injection, the tumors were removed from the mice. The resected tumors were fixed in buffered formaldehyde, embedded in paraffin blocks, and subjected to immunohistochemical and histologic examination.

### *In vitro* differentiation: neural differentiation of marmoset iPS cells

To induce neural differentiation, we cultured marmoset iPS cells using the modified SDIA method (Kawasaki *et al.* 2000). Several colonies of marmoset iPS cells were picked up, partially dissociated, and cocultured with semi-confluent PA6 cells. Cells were plated onto gelatin-coated coverslips at a density of 9 × 10^3^ cells/cm^2^ in Glasgow’s modified Eagle’s medium (GMEM) supplemented with 10% KSR, 2 mm glutamine, 1 mm sodium pyruvate, 0.1 mm nonessential amino acids, and 0.1 mm 2-ME (differentiation medium). The medium was changed every 2 days. On day 17, the differentiation medium was switched to GMEM supplemented with N2, 100 μm tetrahydrobiopterin, 200 μm ascorbic acid, 2 mm glutamine, 1 mm sodium pyruvate, 0.1 mm nonessential amino acids, and 0.1 mm 2-ME. On day 24, the cells were fixed with 4% paraformaldehyde and processed for immunocytochemical analysis with anti-Nestin (Rabbit IgG, 1 : 4000; GenBank Accession No. X65964) and anti-βIII-tubulin (mouse IgG, 1 : 1000; Sigma) antibodies.

### DNA microarray analysis

ES and iPS cell lines were grown on DuplexDish 50 (Carl Zeiss Japan, Tokyo, Japan), fixed with methanol for 3 min at room temperature, and dried by air for 5 min. ES and iPS colonies were microdissected under visual control using the PALM Microbeam System (Carl Zeiss) and catapulted to PALM AdhesiveCaps (Carl Zeiss). Total RNA from 100 to 200 ES and iPS colonies was isolated using the RNeasy Plus Micro kit (QIAGEN). RNA was reverse transcribed, biotin-labeled, and hybridized for 16 h to a Marmoset Genome oligonucleotide custom array Marmo2 (in preparation), which was subsequently washed and stained in a Fluidics Station 450 (Affymetrix Japan, Tokyo, Japan) according to the manufacturer’s instructions. The microarrays were scanned using a GeneChip Scanner 3000 7G (Affymetrix), and the raw image files were converted into normalized signal intensity values using the RMA algorithm (Irizarry *et al.* 2003).

### Hierarchical clustering analysis

Unweighted pair-group method using the arithmetic average analysis was performed using the Euclidian distance to determine the similarity measure and the input rank of all probe sets as the ordering function. Either the complete data or a subset of all the columns constituting the complete data is shown in the figures.

## Supporting Information/Supplementary material

The following Supporting Information can be found in the online version of the article:

**Figure S1** Microsatellite analysis showed complete correspondence between the original liver cells and generated iPS cells, indicating that these iPS cells were certainly generated from liver cells.

**Figure S2** The primer sets we used in this study.

**Figure S3** The iPS cell lines (line B and 1) showed alkaline phosphatase activity (A and G) and expressed SSEA-3 (B and H), SSEA-4 (C and I), TRA-1-60 (D and J), and TRA-1-81 (E and K) but not SSEA-1 (F and L).

**Figure S4** Hierarchical cluster analysis of global gene expression.
