# Supplementary material for: Generating induced pluripotent stem cells from common marmoset (Callithrix jacchus) fetal liver cells using defined factors, including Lin28
Source: Genes Cells. 2010 Sep;15(9):959–69. doi: 10.1111/j.1365-2443.2010.01437.x (PMC2970909; doi:10.1111/j.1365-2443.2010.01437.x)
Supplement: Supplementary file 1 [file gtc0015-0959-SD1.pdf]

Supplemental Figure 1.

	2463-TH-FAM	CJ060-PET	CJ077-VIC	CJ081-VIC	CJ103-NED	CJ187-FAM	CJ003-NED	CJ083-VIC	CJ091-FAM	CJ146-PET
ES cell	109	135	205	164 168	115 123	208	92 100	122 124	139	134
Liver iPS cell	109	135 137	205 211	168 186	115 123 126	201	94 98	122	139	134
Liver cell	109	135 137	205 211	168 186	115 123 126	201	94 98	122	139	134

Supplemental Figure 2.

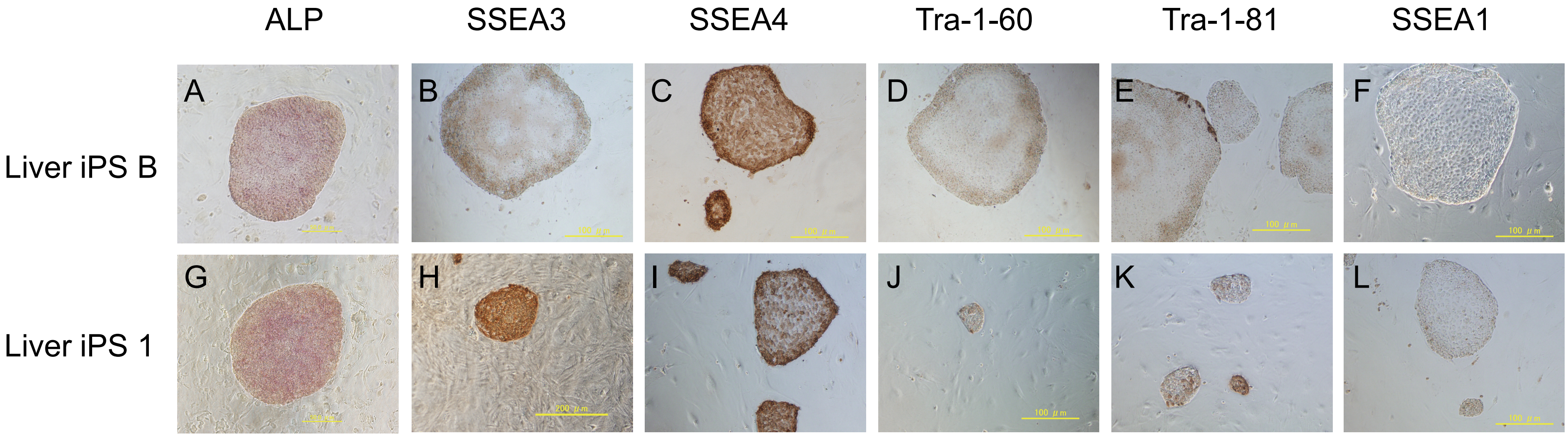

Supplemental Figure 3.

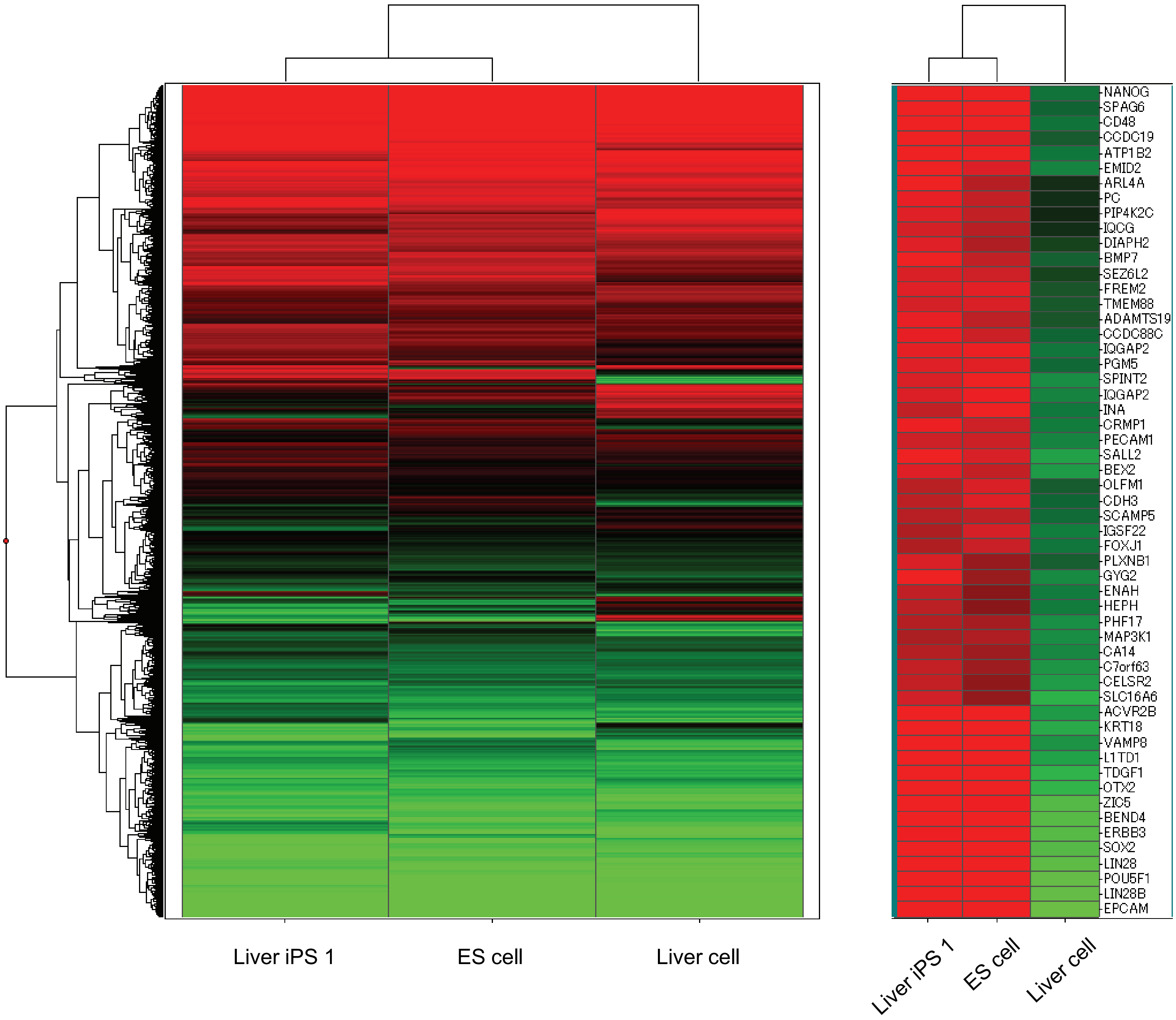

Supplemental Figure 4.

endogenous	Oct-3/4	forward	CCAGGAATCGGGCCAGGGGTTGGGCCAGGC
		reverse	CAGTAAAGGCCGCAGCTTACACATGTTCTT
	Sox2	forward	AATGTTTTAATATTTGCAAGCAACTTTTGT
		reverse	ACAGCTACGGAAAATAAAGGGGGAAAACCT
	Klf4	forward	GGAGCTCTCCCACATGAAG
		reverse	CAGCGAATTGGAGAGGATAA
	c-Myc	forward	ACGCAGCCCCTCCCTCCACGCGGAAGGACT
		reverse	AGTCTCAAGACTCAGCCAAGGTTGTGAGGT
	Nanog	forward	TTCCTTCCTCCATGGATCTG
		reverse	TCCTTGGCCAGTTGTTTTTC
	Lin28	forward	CAACCAACAGTTTGCAGGTG
		reverse	AGATGGATTCCAGACCCTTG
	B-actin	forward	TCCTGACCCTGAAGTACCCC
		reverse	GTGGTGGTGAAGCTGTAGCC
exogenous	Oct-3/4	forward	CCC CAG GGC CCC ATT TTG GTA CC
	Sox2	forward	GGC ACC CCT GGC ATG GCT CTT GGC TC
	Klf4	forward	ACG ATC GTG GCC CCG GAA AAG GAC C
	c-Myc	forward	CCACTGGTCCTCAAGAGGTG
	Nanog	forward	AACAATCAGGCCTGGAACAG
	Lin28	forward	GTCTGGAATCCATCCGTGTC
	GFP	forward	CAAGGACGACGGCAACTACAAGACC
	pMx	reverse	CCC TTT TTC TGG AGA CTA AAT AAA

endoderm	HNF	forward	AGGAACACATGGGAACCAAC
		reverse	CTTGACAATCGTGGCGAC
	AFP	forward	CCTTGTGAAGCAAAAGCCAC
		reverse	CTCCCAAAGCAGCACGAC
	Pdx1	forward	GCCTTTCCCATGGATGAAG
		reverse	CGGTCAAGTTCAGCATTACG
mesoderm	Nkx2.5	forward	CAGAGCTGCGAGCAGAGC
		reverse	GCTGTCCACCTCTGGCTTC
	Gata4	forward	CTCCAGCAGTGCCACCAC
		reverse	CTGGTCTGCGGAGACTGG
	Brachyury	forward	ATGCTCCCCATGAGCCAC
		reverse	CCCTTCGTACAGTGGGGAT
ectoderm	Nestin	forward	GGCTCCAAGACTTCCCTCA
		reverse	CCTGGAGGAGGTCTCGGT
	Sox1	forward	GGGCTCGCTGGTCAAGTT
		reverse	GGAAACGTCTGTACAAAAGCCA
